# A Normalized Tree Index for identification of correlated clinical parameters in microarray experiments

**DOI:** 10.1186/1756-0381-4-2

**Published:** 2011-01-19

**Authors:** Christian W Martin, Anika Tauchen, Anke Becker, Tim W Nattkemper

**Affiliations:** 1 University of Bielefeld, Faculty of Technology, Biodata Mining & Applied Neuroinformatics Group, P.O.-Box 100131, D-33501 Bielefeld, Germany; 2 University of Bielefeld, CeBiTec, Graduate School Bioinformatics and Genome Research, P.O.-Box 100131, D-33501 Bielefeld, Germany; 3 University of Bielefeld, Faculty of Public Health, P.O.-Box 100131, D-33501 Bielefeld, Germany; 4 University of Freiburg, Center for Biological Systems Analysis, FRISYS - AG Becker, Habsburgerstr. 49, D-79104 Freiburg, Germany

## Abstract

**Background:**

Measurements on gene level are widely used to gain new insights in complex diseases e.g. cancer. A promising approach to understand basic biological mechanisms is to combine gene expression profiles and classical clinical parameters. However, the computation of a correlation coefficient between high-dimensional data and such parameters is not covered by traditional statistical methods.

**Methods:**

We propose a novel index, the Normalized Tree Index (NTI), to compute a correlation coefficient between the clustering result of high-dimensional microarray data and nominal clinical parameters. The NTI detects correlations between hierarchically clustered microarray data and nominal clinical parameters (labels) and gives a measurement of significance in terms of an empiric *p*-value of the identified correlations. Therefore, the microarray data is clustered by hierarchical agglomerative clustering using standard settings. In a second step, the computed cluster tree is evaluated. For each label, a NTI is computed measuring the correlation between that label and the clustered microarray data.

**Results:**

The NTI successfully identifies correlated clinical parameters at different levels of significance when applied on two real-world microarray breast cancer data sets. Some of the identified highly correlated labels confirm the actual state of knowledge whereas others help to identify new risk factors and provide a good basis to formulate new hypothesis.

**Conclusions:**

The NTI is a valuable tool in the domain of biomedical data analysis. It allows the identification of correlations between high-dimensional data and nominal labels, while at the same time a *p*-value measures the level of significance of the detected correlations.

## Background

**Figure 1:**
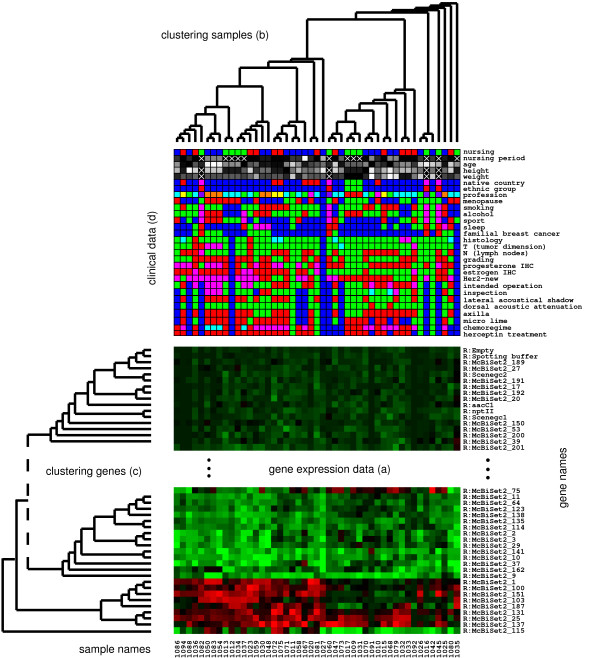
**Visualization of clustered microarray data and clinical data**. The gene expression data (primary data) is displayed by colored squares, each one representing a specific gene and sample. A green square represents an up-regulation, a black square an unchanged expression and a red square a down-regulation compared to reference. The microarray data is clustered both with respect to subjects and genes **(a)**. The hierarchical clustering result is displayed as a tree on the top and on the left side of the data **(b **and **c)**. The rows and columns are permuted according to the leaves of the cluster trees. Clinical data (secondary data) available for the subjects is displayed between the top cluster tree and the microarray data **(d)**.

Hierarchical agglomerative clustering is the basis for most visual data mining tasks in microarray applications [1-3]. Compared to non-hierarchical cluster algorithms, it has the advantage that the number of clusters does not have to be specified in advance. This property is of utmost importance since the number of clusters is usually unknown making a precise a priori prediction of the number of clusters impossible. A second reason for the frequent application of hierarchical agglomerative clustering is its visualization ability [4]. The intrinsic hierarchical cluster structure of the data becomes visually accessible at once in the computed cluster tree. The visualization ability of computed cluster trees is especially valuable to analyze complex biomedical data, consisting of *primary data *and *secondary data*. The primary data is obtained in the main experiment whereas the secondary data includes all supplementary data about the analyzed subjects. In the context of gene expression analysis, the primary data is the gene expression data from the microarray experiments. The corresponding secondary data consists of clinical data, disease outcome, information about the applied treatments and therapies, as well as gene annotations. It is common practice to visualize the computed cluster tree in combination with the clustered microarray data (the primary data) and the secondary data available for the clustered samples (Figure 1).

Microarray technology is currently entering the field of medicine [5,6]. In order to identify molecular factors for macroscopic observations and diseases, recent medical studies often incorporate both gene expression data as well as a high number of clinical parameters [7]. Most mechanisms for development and proliferation of complex diseases (e.g., cancer) are still unknown. It is supposed that many new insights into the mechanisms of diseases can be obtained when the microarray data (primary data) is analyzed in combination with the clinical data (secondary data) consisting of master data, vital data, laboratory data and outcomes (with respect to diseases of interest) that is available for each subject. In few cases, a single gene directly determines the macroscopic phenotype (e.g., eye color). However, most macroscopic phenotypes originate from a set of genes, denoted as gene profiles or metagenes [8]. Clinical data can be considered as a set of observations on the phenotypic level. There are observations on the molecular level (e.g., protein expression), macroscopic observations (e.g., skin color, tumor size, outcome) as well as behavioral observations (e.g., nutrition, alcohol consumption, sport). One issue of interest to the researcher is the identification of clinical parameters (labels) that are correlated with the microarray data. A high correlation between a label and the microarray data indicates that there might be a common underlying mechanism or pathway. This provides a good basis to formulate new hypothesis and to obtain new insights into the complex mechanisms of diseases.

The visual inspection of cluster trees allows the estimation of the correlation between the label and the clustered microarray data. However, this approach becomes infeasible for studies with large numbers of samples and a high number of different labels. Furthermore, the number of labels available for each subject is continuously increasing, since hospital information systems store large amounts of laboratory and vital data as well as radiological and microbiological findings in huge databases [9]. Therefore, an automated and objective computation of the correlation between labels and microarray data is needed to identify correlated clinical parameters.

The canonical way to compute the correlation between a label and the microarray data is to compute the correlation between the label (first variable) and every single gene (second variable), and to combine the results in a final correlation coefficient. Depending on the type of variables, statistics provides various methods to compute the correlation between two variables. For interval data, Pearson's correlation coefficient *r *[10] computes the correlation between two variables whereas each variable is normalized to zero mean and unit variance beforehand. For ordinal data, the correlation between two variables can be computed using Spearman's rank correlation coefficient *ρ *[11]. This robust measure can even be applied on small sample sizes, but it requires that the original data of any two successive ranks has to be approximately equidistant. In cases where this can not be assumed, Kendall's *τ *[12] should be used instead. For nominal data, the chi-square test, Pearson's contingency coefficient, or the corrected contingency coefficient measure the correlation between any two variables [11].

A major drawback of the different correlation and contingency coefficients is that they can only be used to compute the correlation between a label and a single gene. Information contained in metagenes or gene profiles cannot be assessed this way. Thus, a direct computation of the correlation between a label and single genes in order to identify correlated labels does not capture the major trend of information hidden in the data. Microarray data rather has to be considered in its entirety, and an analysis always has to be done in an holistic way.

In this paper, we propose a novel index, the Normalized Tree Index (NTI), which is an extension of the Tree Index (TI) proposed in [13]. The NTI computes a correlation coefficient between the clustering result (tree structure) of high-dimensional primary data (here: microarray data) and associated nominal labels of secondary data (here: clinical parameters). Due to a normalization procedure it is bounded by [0, 1]. A high NTI indicates a high correlation between the label and the clustered data and vice versa.

**Figure 2:**
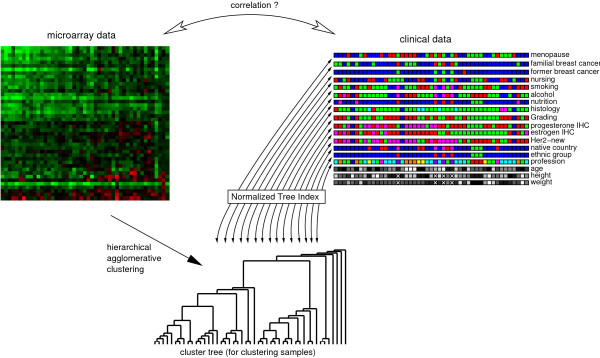
**Computing the correlation between clinical parameters and microarray data**. The Normalized Tree Index (NTI) is used to compute a correlation coefficient between the clustering result (tree structure) of high-dimensional primary data (microarray data) and associated nominal labels of secondary data (clinical parameters). In a first step, the microarray data is clustered by hierarchical agglomerative clustering using standard settings. In a second step, the computed cluster tree is evaluated using the NTI. For each label, a NTI is computed that measures the correlation between that label and the clustered microarray data. This permits the identification of labels that are highly correlated with the microarray data, while analyzing the microarray data in its entirety.

Furthermore, an empirical *p*-value is derived which measures the level of significance of the detected correlations between labels and clustered microarray data. In a first step, the microarray data is clustered by hierarchical agglomerative clustering using standard settings (Figure 2). Thereby, the complete microarray data is taken into account. In a second step, the computed cluster tree is evaluated using the NTI. For each label, one NTI is computed measuring the correlation between that label and the clustered microarray data. By this approach the microarray data is considered in its entirety and labels that are correlated with the microarray data can be identified. The NTI extends the TI in many respects: First, the normalization procedure increases the interpretability of the correlation result considerably. The TI has been biased with respect to the number of classes of the label, the number of elements of each class, and the number of missing values. This unwanted feature prevents an objective correlation analysis with different labels whose number of classes vary. Second, the computation of the *p*-value: The *p*-value is a valuable parameter for the biomedical researcher since it measures the level of significance of any detected correlation. Both the normalization and the *p*-value enables us to automatically detect correlations between clustered genomic data (primary data) and many different clinical parameters (secondary data). Thus, the scope of this paper is far beyond that of [13], which was to improve the clustering process for one fixed clinical parameter by detecting the most appropriate parametric setting to obtain the best clustering result. In this paper, we aim to discover new relationships between genomic data and macroscopic observations. We rather focus on knowledge discovery in data bases (KDD) than on a pure data mining task.

The NTI is successfully applied on two breast cancer data sets to compute correlations between microarray data and clinical data. Some of the identified highly correlated labels confirm the actual state of knowledge in breast cancer research (i.e. progesterone IHC, estrogen IHC). Others are helpful to identify new risk factors and provide a good basis to formulate new hypothesis and to obtain new insights into the complex mechanisms and pathways of diseases.

## Methods

Cluster indices are cluster validation techniques that provide an objective measure of a clustering result. They can be grouped into *internal *and *external *ones [14-16]. Internal cluster indices evaluate the quality of a clustering result by using only intrinsic information of the data. In contrast to that, external cluster indices permit an entirely objective evaluation by making use of the knowledge of an external class label, denoted as *label *in the following. The Tree Index (TI) is an external cluster index for cluster trees [13]. It is used to identify the algorithm and parameterization yielding the clustering that is best suited for visualization. However, the TI has the drawback that it is biased with respect to the number of classes of the label, the number of elements of each class, and the number of missing values. This unwanted feature prevents an objective correlation analysis with different labels whose number of classes vary. To overcome this problem, an extension to the TI, the Normalized Tree Index (NTI), is developed. The Normalized Tree Index (NTI) computes a normalized correlation coefficient between hierarchically clustered primary data (microarray data) and nominal labels of secondary data (clinical parameters). Furthermore, a *p*-value is derived that measures the level of significance of the detected correlation between labels and clustered data. The NTI and the corresponding *p*-value are computed for each label of the secondary data.

### The Tree Index (TI)

Let the primary data be a dataset X of *d *samples of length *g*: X = {**x**_1_, ..., **x***_i_*, ..., **x***_d_*}, length (**x***_i_*) = *g*. In the context of microarray data analysis, X can be a preprocessed microarray data set with *d *tissue samples and *g *genes. A label **c **(dim (*c*) = *d*) is selected from the secondary data for correlation analysis (e.g., grading), with $c_{i}\in \{C_{1},....,C_{k}\}$, *i *= 1, ..., *d *and *κ *the number of classes (e.g., the number of gradings). Let X be clustered by hierarchical agglomerative clustering. After the clustering, the TI is computed for each label on the resulting cluster tree.

The *Tree Index (TI) *considers the cluster tree as a result of a statistical splitting process. It is based on the evaluation of probabilities of every single split in the tree starting from the root (i.e. the entire dataset is one cluster). In a first step, a *splitting score *is computed for every single split in the tree based on the probability of the split. In a second step, all splitting scores are combined to compute the final TI.

*Step 1 *Let the cluster of the *r*-th split (the splits are numbered arbitrarily) contain *N *elements. Let the cluster be split into *l *(usually *l *= 2) smaller subclusters. The elements of the main cluster belong to *κ *different categories whereas *n*_λ_, λ ∈ {1, ..., *κ*} specifies the number of elements belonging to class $C_{\lambda }$. The *i*-th subcluster contains *m_i _*elements with *m*_*iλ *_elements belonging to class $C_{\lambda }$. The primary objective is to compute the probability of such a particular split by taking the observed class distributions in the clusters into account. It is assumed that *m*_*i*, _*i *∈ {1, ..., *l*} elements are drawn from the *N *elements by sampling without replacement. Thereby each element is drawn with the same probability. The probability of the observed class distribution in the splitted clusters is given by a generalized form of the *polyhypergeometric distribution *or *multivariate hypergeometric distribution *[17]. Let **M **= {*m*_*iλ*_}, **n **= {*n*_λ_}, and **m **= {*m_i_*} with 1 ≤ *i ≤ l *and 1 ≤ λ *≤ κ*.

(1) $$p(M;N,n,m)=\frac{\prod_{i=1}^{l}\frac{m_{i}!}{\Pi_{\lambda =1}^{\kappa }m_{i\lambda }!}}{\frac{N!}{\Pi_{\lambda =1}^{\kappa }n_{\lambda }!}}$$

*p*(**M**; *N*, **n**, **m**) decreases with the size of the cluster that is split and with the homogeneity of the subclusters. The splitting score *S_r _*of the *r*-th split is defined by its negative logarithmic probability.

(2) $$\begin{array}{c} S_{r}(M;N,n,m)\;=\;-\ln p(M;N,n,m)\\ =\ln N!\;-\sum_{\lambda =1}^{\kappa }\ln\;n_{\lambda }!\;-\sum_{i=1}^{l}\left(\ln m_{i}!\;-\sum_{\lambda =1}^{\kappa }\ln\;m_{i\lambda }!\right) \end{array}$$

**Figure 3:**
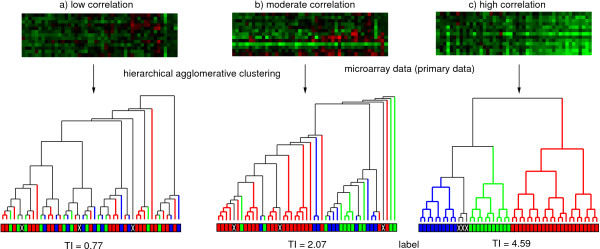
**Correlation between different microarray data sets and a label**. The correlation between three different microarray data sets and a label is analyzed using the TI. In **(a)**, there is only a low correlation between the microarray data and the label, resulting in a TI of 0.77. A TI of 2.07 indicates a moderate correlation in **(b)**, whereas a high correlation (TI = 4.59) is shown in **(c)**.

*Step 2 *The TI combines the complete set of splitting scores to a parameter-free index by computing the standard deviation of splitting scores:

(3) $$\text{T1=}\sqrt{\frac{1}{R}\sum_{r=1}^{R}{\left(S_{r}-\bar{S}\right)}^{2}},\;\text{with}\;\bar{S}=\frac{1}{R}\sum_{r=1}^{R}S_{r},$$

and *R *being the number of splits in the cluster tree. The higher the index for a given label, the higher the correlation between that label and the clustered primary data (Figure 3). As stated earlier in this section, the TI is biased with respect to the number of classes of the label. If the number of classes of the label increases, the TI also increases. This is due to the fact, that *p*(**M**; *N*, **n**, **m**) decreases if the number of classes and thus the number of possible class distributions increases. This leads to higher splitting scores and thus a higher TI. For a more detailed description of the TI, please refer to [13].

### The Normalized Tree Index (NTI)

**Figure 4:**
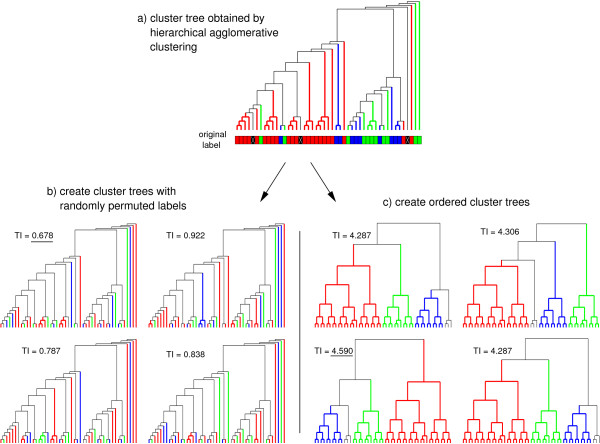
**Lower and upper bound for the TI**. The cluster tree that is obtained when applying hierarchical agglomerative clustering to the primary data is shown in **(a)**. The branches and leaves of the cluster tree are colored according to the given label. Missing values are colored in black. In **(b) **the labels are permuted *r *times. The cluster tree with the lowest TI (underlined) is an empirical estimation for TI_min_. The TIs are also used to compute the respective *p*-value. In **(c) ***r *ordered cluster trees and ordered labels are generated. The cluster tree with the highest TI (underlined) is an empirical estimation for TI_max_.

The Normalized Tree Index (NTI) computes a normalized correlation coefficient between nominal parameters and hierarchically clustered data. In order to avoid biases with respect to the number of classes and the distribution of cluster sizes [14,15], the TI is normalized as suggested in [15]. It should be noted that this normalization procedure does not lead to an unbiased correlation coefficient in a strong statistical sense. The distribution of the TI for different number of classes and cluster sizes is not taken into account. After the normalization, the expectation *E*[NTI] is still unknown. We propose to empirically calculate TI_min _and (TI_max_) for each considered label by using a Monte Carlo simulation (Figure 4):

To compute TI_min_, the labels are permuted *r *≥ 10000 times (Figure 4b), whereas the cluster tree remains unchanged. For each randomly permuted label and each random cluster tree, a TI is computed. The lowest TI is an empirical estimation for TI_min_.

To compute TI_max_, *r *≥ 10000 ordered labels and ordered cluster trees are generated (Figure 4c). An ordered cluster tree consists of *κ *pure subtrees, each one containing all items belonging to one class. The internal structure of each pure subtree is of no importance and is chosen arbitrary. Based on the *κ *pure subtrees, the ordered tree is constructed by merging two randomly selected subtrees in *κ *- 1 agglomerative steps. The ordered label is constructed respectively. For each ordered label and each ordered cluster tree, a TI is computed. The highest TI is an empirical estimation for TI_max_. With the estimations for TI_min _and TI_max_, the NTI is defined by

(4) $$\text{NTI}=\frac{\text{TI}-{\text{TI}}_{\min}}{{\text{TI}}_{\text{max}}-{\text{TI}}_{\min}}$$

The NTI is bounded by [0, 1]. However, the empirical computation of TI_min _and TI_max _implies that there might be a TI < TI_min _or a TI > TI_max_. In such a case, the NTI should be set to 0 or 1, respectively. To reduce the probability for such events, *r *should be set sufficiently large.

A high NTI indicates a high correlation between the label and the clustered data and vice versa.

### Computation of *p*-value

Natural fluctuation in the data can lead to constellations in which the clustered data seems to be correlated with external labels, but in fact the correlation has occurred by chance. The computation of a *p*-value allows the detection of such false identifications of correlations. This approach has already been successfully applied for the biological homogeneity index (BHI) and the biological stability index (BSI) [18].

Let *H*_0 _be the null hypothesis that there is no correlation between the microarray data and a clinical parameter. A *p*-value lower than a significance level of 5%, 1%, or 0.1% means a rejection of *H*_0_. The *p*-value can either be derived analytically or empirically. Here, a Monte Carlo simulation is used to compute an empirical *p*-value for the TI and NTI. For simplicity, the computation of the *p*-value is derived for the TI.

**Figure 5:**
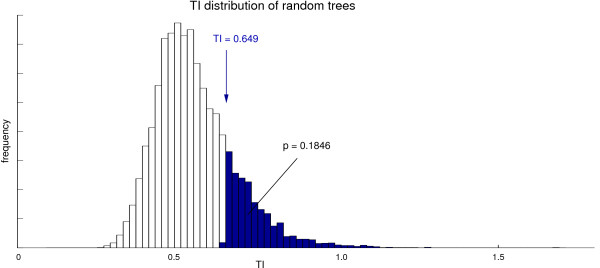
**Computation of an empirical ***p***-value for the TI and NTI**. An empirical *p*-value for the TI (as well as for the NTI) is obtained using a Monte Carlo simulation: A TI is computed *r *≥ 10000 for the same clustered tree but randomly permuted labels. The *p*-value is defined by the fraction of TIs that is equal or higher than the TI obtained from the original label and cluster tree (here: *p *=18.46%). The resulting *p*-value of 0.1846 ≥ 0.05 indicates that there is no significant correlation between the clustered data and the label.

Let *t *be the TI of the tree obtained by a hierarchical cluster algorithm (e.g., hierarchical agglomerative clustering). The empirical *p*-value is defined by the fraction of TIs obtained from trees with randomly permuted labels (Figure 4b) that is equal or higher than *t *(Figure 5):

(5) $$p=\int_{u\geq t}^{\infty }du$$

For practical use, *p *can be approximated by

(6) $$p\approx \frac{1}{r}\sum_{\begin{array}{l} i=1\\ t_{i}>t \end{array}}^{r}1$$

with *t_i _*being the TI of the clustered tree with the *i*-th randomly permuted label. A *p *≥ 0.05 means that *H*_0 _(no correlation) cannot be rejected. A *p *< 0.05 (0.01, 0.001) means that the rejection of *H*_0 _is statistically significant at the 5% (1%, 0.1%) level. The number *r *of randomly permuted labels has to be sufficiently large to obtain a statistical significant rejection of *H*_0 _at the 5% (1%, 0.1%) level. *r *> 1000 random trees are required to theoretically achieve a statistical significant rejection of *H*_0 _at the 0.1% level. Therefore consider the extreme example that *r *random trees are computed and that each *t_i _*is lower than *t*. This results in the empirical *p*-value of 1/*r*. Thus, *r *> 1000 random trees are required to achieve a *p*-value of less than 0.001 and a rejection of *H*_0 _at the 0.1% level.

There is no need to use random trees instead of the clustered tree when computing the *p-*value. If random trees *and *randomly permuted labels were used, two variables would be randomized at the same time. This would lead to an artificially inflated search space. With the computation of the *p*-value, we measure the significance of the correlation between the clustering and the categorical clinical classes. The intention is not to determine if there is both a significant correlation between the clustering and the categorical clinical classes *and *a significant clustering.

The *p*-value is not altered by the normalization. It is equal for the TI and the NTI. This is due to the fact that the tree indices are only shifted and scaled in Equation 4. Thereby, the fraction of *t_i _*>*t *remains unchanged.

*Example *Let us assume that hierarchical agglomerative clustering of some primary data leads to a TI of *t *= 0.688 (Figure 5). Let us further assume that the Monte Carlo simulation reveals that 18.46% of TIs obtained from ordered cluster trees and randomly permuted labels are higher than *t*. The resulting *p*-value of 0.1846 indicates that *H*_0 _(no correlation) cannot be rejected at the 5% level of significance.

## Results

The NTI and *p*-value is applied on two microarray breast cancer data sets. The first data set is the breast cancer data set of van de Vijver [19] (downloadable at [20]), which is an extension to the study of van't Veer [7]. For each of the 295 subjects, 24496 genes are analyzed and six nominal clinical parameters are available (Table 1). The clustering of subjects is performed on logarithms of a set of 231 marker genes (identified in [7]). The second data set is a preliminary data set taken from the Bielefeld breast cancer project (BBCP) [21,22]. In the BBCP, a set of 201 marker genes is analyzed for 87 samples taken from 49 patients. One main feature of the BBCP, in contrast to other microarray breast cancer studies, is the high number of clinical parameters that has been collected for each of the patients. As summarized in Table 2 (left column), 29 clinical parameters are selected for correlation analysis. Some of these parameters are interval parameters. To apply the NTI, they have to be converted to nominal parameters by parameter-specific transformations (e.g., the values of the *body mass index *(BMI) are divided into the three categories *normal *(18.5 to 25), *overweight *(25 to 30), and *obese *(> 30). Moreover, if reasonable, the categories of some nominal variable are merged (e.g., the categories of the parameter *progesterone receptor IHC *are transformed to the categories *negative *(for values 0 to 1), *intermediate *(for values 2 to 8) and *high positive *(for values 9 to 12)). All specific transformations are listed in Table 2 (right column).

**Table 1:** Clinical parameters of the van de Vijver data set

clinical parameter	num	categories
metastasis	2	(1) no (2) yes
positive lymph nodes	2	(1) no (2) yes
event death	2	(1) no (2) yes
estrogen receptor	2	(1) negative (2) positive
National Institute Health criteria	2	(1) 0 (2) 1
St. Gallen consensus criteria	2	(1) 0 (2) 1
conservative flag	3	(1) 0 (2) 1 (3) 2

The clinical parameters and their categorizations in the van de Vijver breast cancer data set.

**Table 2:** Clinical parameters of the BBCP data set

clinical parameter	num	categories
age	6	(1) <40 (2) 40 to 49 (3) 50 to 59 (4) 60 to 69 (5) 70 to 79 (6) > 79

sample type	3	(1) biopsie before chemotherapy (2) biopsie after chemotherapy (3) operation (after chemotherapy)

BMI	3	(1) normal (18.5 to 25) (2) overweight (25 to 30) (3) obese (> 30)

native country	6	(1) Germany (2) Poland (3) Russia (4) Taiwan (5) Sri Lanka (6) Turkey

ethnic group	2	(1) Europe (2) Asia

nursing	2	(1) no (2) yes

nursing period	4	(1) none (2) short (1 to 5 months) (3) intermediate (6 to 14 months) (4) long (> 14 months)

menopause	2	(1) no (2) yes

smoking	5	(1) always non-smoker (2) sometimes (3) regular (4) often (5) again non-smoker

alcohol	5	(1) never (2) no longer (3) less than once a month (4) 1 to 3 times a week (5) daily

sport	3	(1) nothing (0 h/week) (2) little (1 to 4 h/week) (3) plenty (> 5 h/week)

sleep	3	(1) little (< 7 h/day) (2) normal (7-9 h/day) (3) plenty (> 9 h/day)

familial breast cancer	2	(1) No (2) yes

histology	4	(1) ductal (2) lobar (3) not definable (4) mucous ductal

T (tumor dimension)	5	(1) T0 (2) T1 (3) T2 (4) T3 (5) T4

N (lymph nodes)	3	(1) N0 (2) N1 (3) N2

Grading	2	(1) G2 (2) G3

Progesterone receptor IHC	3	(1) negative (0 to 1) (2) intermediate (2 to 8) (3) high positive (9 to 12)

Estrogen receptor IHC	3	(1) negative (0 to 1) (2) intermediate (2 to 8) (3) high positive (9 to 12)

Her2-new	3	(1) negative (2) intermediate (3) positive

intended operation	5	(1) ablatio and axilla (2) ablatio and sentinel (3) BET and sentinel (4) ablation (5) BET

inspection	4	(1) no conspicuity (2) in ammatory mamma-carcinoma (3) plateau phenomenon (4) other

lateral acoustical shadow	2	(1) no (2) yes

dorsal acoustic attenuation	2	(1) no (2) yes

axilla	2	(1) unsuspicious (2) suspicious

tumor size (mammogramm)	3	(1) small (0 to 9 mm) (2) intermediate (10 to 25 mm) (3) large (> 26 mm)

micro lime	2	(1) no (2) yes

chemoregime	5	(1) TAC (2) ACDoc (3) Geparquattro (4) FEC (5) Geparquinto

herceptin treatment	2	(1) no (2) yes

The clinical parameters and their categorizations in the BBCP data set: Interval parameters are converted to nominal parameters by the indicated transformations in parentheses.

In this paper, the entire molecular expression signature is used to demonstrate the merits of the NTI and its *p*-value. Available knowledge about the analyzed genes, i.e. information about marker genes, is used anyway since this helps to create well-structured cluster trees. Both data sets (van de Vijver and BBCP) are preprocessed and clustered as follows: The logarithms of ratios between the respective gene expression to reference sample are scaled to [-1, 1]. Let **x***_st _*be the scaled logarithm of sample *s *and gene *t*. The expression profiles **x***_s _*are clustered by hierarchical agglomerative clustering using average linkage and a distance metric (dissimilarity measure) based on the correlation between a pair of subjects. This correlation distance metric *d_ij _*∈ [0, 1] of two expression profiles **x***_i _*and **x***_j _*of length *g *is defined as

(7) $$d_{ij}=\frac{1}{2}-\frac{\sum_{k=1}^{g}x_{ik}x_{jk}}{2g}$$

By applying the NTI on cluster trees obtained from real-world data sets, we simulate the scenario where a biomedical researcher is looking for clinical parameters that are correlated with the microarray data. The NTI and *p*-value are computed for each clinical parameter listed in Tables 1 and 2. This enables the detection of even unexpected relations between the variables. By this approach, huge data collections can be screened without the requirement to manually pre-select the clinical parameters. Nevertheless, the insight gained depends on the parameter, e.g. the parameter *intended operation *rather reveals an unexpected relationship than any insight into a biomedical process.

**Figure 6:**
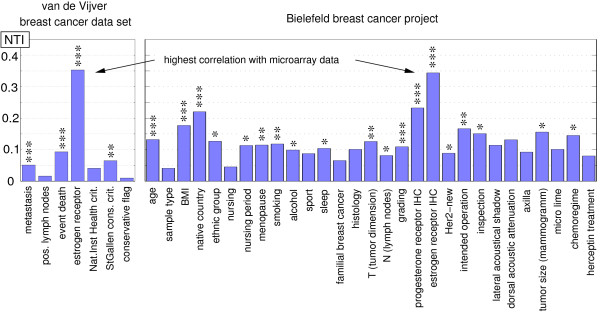
**Summary of NTIs for the van de Vijver and the BBCP data set**. A NTI and *p*-value is computed for each clinical parameter. For both data sets, the highest NTI is obtained for the estrogen receptor. The number of asterisks indicates the level of significance of the correlation. One asterisk means that the rejection of *H*_0 _(no correlation) is statistically significant at the 5% level, two asterisks at the 1% level, and three asterisks at the 0.1% level. No asterisk means that H_0 _cannot be rejected.

A summary of all results is shown in Figure 6. For both data sets, the highest NTI is obtained for the estrogen receptor. The number of asterisks indicates the level of significance of the correlation. One asterisk means that the rejection of *H*_0 _(no correlation) is statistically significant at the 5% level, two asterisks at the 1% level, and three asterisks at the 0.1% level. No asterisk means that *H*_0 _cannot be rejected.

On the left of Figure 6, the results for the van de Vijver breast cancer data set are shown. The highest NTI is obtained for the clinical parameter *estrogen receptor (ESR1)*. The correlation between the clinical parameters *metastasis*, *event death*, and *estrogen receptor (ESR1) *and the microarray data is statistically significant at the 0.1% level. The *StGallen consensus criteria *is statistically significant at the 1% level. On the right of Figure 6, the results for the BBCP data set are shown. The highest NTI is obtained for *estrogen receptor IHC*. The correlations between *age*, *BMI, native country, grading, progesterone receptor IHC*, and *estrogen receptor IHC *and the microarray data are statistically significant at the 0.1% level. The correlations of the parameters *menopause, smoking*, *T *(*tumor dimension*), and *intended operation *are statistically significant at the 1% level. The correlations of the parameters *ethnic group, nursing period, alcohol, sleep, N (lymph nodes)*, *Her2-new, inspection, tumor size (mammogramm*) and *chemoregime *are statistically significant at the 5% level.

**Figure 7:**
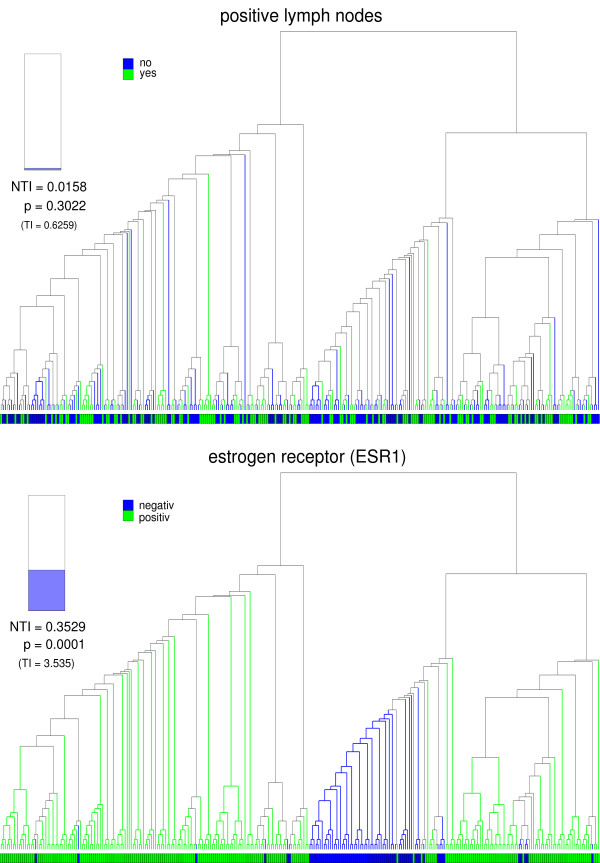
**Cluster tree of the van de Vijver data set**. The cluster tree obtained from the van Vijver data set is colored and evaluated with respect to the clinical parameters *positive lymph nodes *and *estrogen receptor (ESR1)*. The *p*-value of 0.3022 indicates that the microarray data is not correlated with *positive lymph nodes*. In contrast to that, the microarray data is highly correlated with *estrogen receptor (ESR1)*.

**Figure 8:**
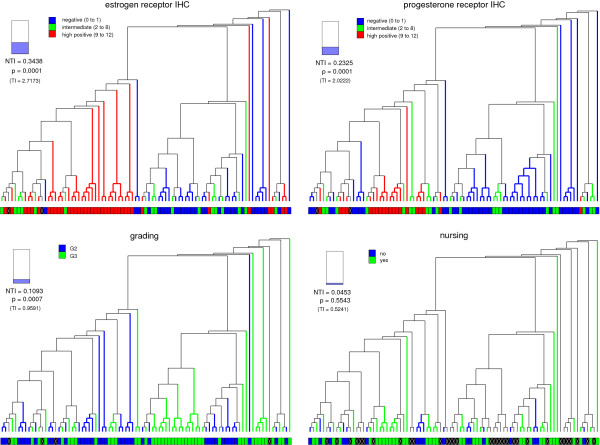
**Cluster tree of the BBCP data set**. The cluster tree obtained from the BBCP data set is colored and evaluated with respect to the clinical parameters *estrogen receptor IHC, progesterone receptor IHC, grading*, and *nursing*. The *p*-values < 0.001 indicate that the microarray data is highly correlated with the first three parameters, whereas there is no correlation with *nursing*.

The cluster tree of the van de Vijver data set is displayed in Figure 7. It is colored and evaluated with respect to the clinical parameters *positive lymph nodes *and *estrogen receptor *(*ESR1*). No correlation is detected between the microarray data and *positive lymph nodes*. In contrast to that, the microarray data is highly correlated with *estrogen receptor (ESR1)*, since the rejection of *H*_0 _is statistically significant at the 0.1% level. The cluster tree of the BBCP data set is displayed in Figure 8. It is colored and evaluated with respect to the clinical parameters *estrogen receptor IHC*, *progesterone receptor IHC*, *grading*, and *nursing*. The *p*-values < 0.001 indicate that the microarray data is highly correlated with the first three parameters, whereas there is no correlation with *nursing*.

## Discussion

A novel index, the Normalized Tree Index (NTI), is developed to compute a normalized correlation coefficient between hierarchically clustered primary data (microarray data) and nominal labels of secondary data (clinical parameters). The NTI is an extension to the TI as described in [13], but it is bounded by [0, 1]. A high NTI indicates a high correlation between the label and the clustered data and vice versa. Furthermore, an empirical *p*-value is derived which measures the level of significance of the detected correlations between labels and primary data.

Some of the detected correlations reflect common knowledge: The clinical relevance of the *estrogen receptor *and the *progesterone receptor *is unquestioned [23-25]. The gene expression of these receptors are main criteria to differentiate between genetic profiles [26-28]. Also, a correlation to *metastasis *and *event death *is shown in [7], and a correlation to *grading *is reported in [22]. For other detected correlations there is no clear evidence provided in the literature: The parameters *age, BMI, native country, menopause, smoking, T (tumor dimension) *are highly correlated with the genomic data. These high correlations indicate that there might be common underlying mechanisms or pathways. The linkages between the phenotypes (the clinical parameters) and the genotypes (the microarray data) help to formulate new hypothesis and aid to obtain new insights into the complex mechanisms of diseases. Some of the detected correlations are harder to interpret: The correlations between *intended operation*, *and chemoregime *and the microarray data are probably not based on direct causal relationships. Interestingly, no significant correlation is reported between the parameters *familial breast cancer, histology, lateral acoustical shadow, dorsal acoustic attenuation *and the microarray data - an indicator that the genomic information offers a new approach to access and thus improve the diagnoses of breast cancer.

Even though applied to microarray data in a medical setting, the NTI can be applied to any complex data, in whose context a cluster analysis of the primary data is reasonable. Whenever there is the slightest assumption that the internal structure of the primary data might be correlated with a given label of the secondary data, the NTI provides an objective measure for this structural relationship.

The Normalized Tree Index (NTI) is developed to compute a correlation coefficient between primary data and nominal labels of secondary data. Ordinal and interval labels have to be converted to nominal labels by label-specific transformations (Table 2). The correlation result depends on the specific transformation set up by the researcher. Even though different categorizations for the labels could be tested this way, background knowledge is required for this step. The transformations also imply a loss of information. However, a reduction of the data of an ordinal or interval parameter to a few biological relevant categories can also help to avoid over fitting. In Table 2, the interval-scaled clinical labels *progesterone receptor IHC *and *estrogen receptor IHC *have been transformed to nominal labels with three categories: negative (0 to 1), intermediate (2 to 8) and high positive (9 to 12). Nevertheless, strategies for a direct application of the NTI on ordinal and interval labels need to be developed.

Hierarchical agglomerative clustering and the computation of the NTI are advantageous compared to the following method that is sometimes used to obtain a correlation coefficient: A classifier is trained on the microarray data. A selected label is used to rate the correlation depending on the ability of a classifier to predict the correct label in a leave-one-out setting. The higher the classification rate, the higher the correlation between the primary data and the label. The major drawback of this approach is that a visualization is not provided this way. A high classification rate indicates a high correlation, but there is no way to retrace how the specific classification rate has been obtained. Homogeneous clusters, outliers, and other significant patterns cannot be identified this way.

## Conclusion

The Normalized Tree Index (NTI) is the first cluster index that uses the structure of the hierarchical clustering tree to compute a normalized correlation coefficient between nominal labels and high-dimensional primary data. Its normalization feature enables the easy identification of labels that are correlated with the primary data, while at the same time a *p*-value measures the level of significance of the detected correlations. Even though applied to microarray data in a medical setting, the NTI can be applied to any complex data. This general applicability makes it a powerful tool in diverse domains.

## Competing interests

The authors declare that they have no competing interests.

## Authors' contributions

CM conceived of and carried out the study and the statistical analysis and drafted the manuscript. AT carried out the expression profiling experiments and the primary data analysis. AB contributed to the design of the experiments and constructed the microarray. TN participated in the design and coordination of the study and in drafting the manuscript. All authors read and approved the final manuscript.
